# Energetic Landscape of MDM2-p53 Interactions by Computational Mutagenesis of the MDM2-p53 Interaction

**DOI:** 10.1371/journal.pone.0147806

**Published:** 2016-03-18

**Authors:** Kelly M. Thayer, George A. Beyer

**Affiliations:** 1 Department of Chemistry, 124 Raymond Avenue, Poughkeepsie, New York 12604, United States of America; 2 Biochemistry Program, Vassar College, 124 Raymond Avenue, Poughkeepsie, New York 12604, United States of America; 3 Wesleyan University, Hall Atwater Laboratories, Middletown, Connecticut 06459, United States of America; Virginia Commonwealth University, UNITED STATES

## Abstract

The ubiquitin ligase MDM2, a principle regulator of the tumor suppressor p53, plays an integral role in regulating cellular levels of p53 and thus a prominent role in current cancer research. Computational analysis used MUMBO to rotamerize the MDM2-p53 crystal structure 1YCR to obtain an exhaustive search of point mutations, resulting in the calculation of the ΔΔG comprehensive energy landscape for the p53-bound regulator. The results herein have revealed a set of residues R65-E69 on MDM2 proximal to the p53 hydrophobic binding pocket that exhibited an energetic profile deviating significantly from similar residues elsewhere in the protein. In light of the continued search for novel competitive inhibitors for MDM2, we discuss possible implications of our findings on the drug discovery field.

## Introduction and Background

MDM2 plays a critical role in understanding cancer and development of novel therapeutics because of the crucial role it plays in the regulation of p53[1]. The tumor suppressor protein p53 acts to suppress tumor growth [2] as originally elucidated in mouse models [3][4][5]. As a transcription factor, p53 acts as the “gatekeeper” of the human genome by effecting DNA repair of apoptosis prior to replication when DNA has incurred damage [2][6][7]. In turn, p53 itself is subject to regulation. One of those regulators, MDM2, negatively regulates p53 via three principle mechanisms [8][9]. It prevents p53 from operating by mediating the cellular export of p53 [10]. As an E3 ubiquitin ligase, it negatively regulates p53 by tagging its carboxy terminus with ubiquitin to mark it for degradation by the proteasome [9][11][12][13]. Furthermore, by interacting with p53’s N-terminal transcription activation domain with an unbinding energy measured at -8.4 kcal/mol [14], as captured in a crystal structure[15], MDM2 directly inhibits transcription [16][17], which is the mechanism frequently targeted by the development of competitive inhibitors. Disruptions interfering with homeostatic regulatory balance causing excessive downregulation of p53 renders cells unequipped to effectively prevent tumor growth; thus, interruptions to the proper regulation between MDM2 and p53 have been associated with a variety of cancers, most notably those in which wild type p53 remains intact [18][19][20][21][22][23][24]. The operative hypothesis suggests that treating hyperactive MDM2 can be addressed by the development of a competitive inhibitor for the p53 transcription activation substrate binding site on MDM2 to decrease the rate at which p53 becomes inactivated. Proof of concept was demonstrated in cell culture by the overexpresson of a peptide homologue of p53, which led to higher cellular activity of p53, which was able to activate downstream effectors and carry out cell cycle arrest and cell death, supporting the idea that disruption of the MDM2-p53 interaction would be sufficient to remedy the normal functionality of p53 and that this constitutes a logical strategy for the development of therapeutics [25]. This premise has prompted research that aims to understand the p53-MDM2 interaction interface [26][27] to inform the discovery of inhibitors [28][29] in hopes of ultimately preventing tumor development in patients who suffer from cancers arising from hyperactive MDM2 activity.

**Fig 1:**
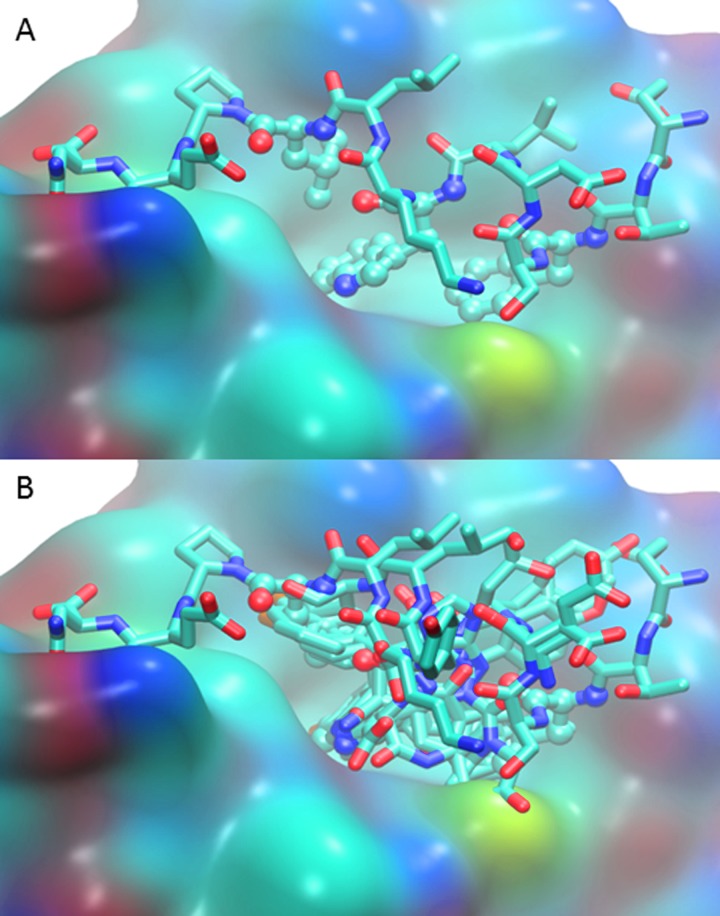
(A) MDM2 binding interface (surface view with CPK atom coloring) with native p53 N-terminal peptide (licorice, also CPK coloring) bound in 1YCR crystal structure [15]. The three key binding residues, Phe19, Trp23, and Leu26, are highlighted with ball and stick view. (B) MDM2-bound p53 N-terminal peptide aligned with representative protein-bound inhibitors. For clarity the protein surface of only 1YCR is shown. The PDB ID and inhibitors included are 1YCR native p53 peptide [15], 1T4E benzodiazepinedione [33], 3LBL MI-63-analog [34], 3LBK imidazol-indole [34], 3JZK chromenotriazolopyrimidine [35], 4HG7 nutlin-3a [36], 4JRG pyrrolidine carboxamide [37], 4UMN stapled peptide [38].

Characterization of the interface between MDM2 and p53 has greatly contributed to the development of high potency therapeutics designed to meet the challenge of disrupting the interaction between MDM2 and p53 via competitive inhibition. At this interface, a hydrophobic region of the MDM2 N-terminus sequesters the N-terminal amphipathic helix of p53, as has been captured by the 1YCR crystal structure[15]. The p53 residues Phe19, Trp23, and Leu26 reach into a hydrophobic pocket of MDM2, and the epsilon nitrogen of Trp23 hydrogen bonds with Leu54 of MDM2 [15] (**Fig 1A**). To shed light on the energetics at play in the interface, alanine scanning has been employed [27]. MDM2 also was one of the first proteins to be analyzed with alanine scanning mutagenesis and subsequent MM-PBSA calculations, which identified key mutable sites along the p53-MDM2 transactivation interface [28][30], and, not surprisingly, included the three directly interacting residues from p53, as well as residues contributed from MDM2 **(Table 1)**. Non-alanine mutations were explored selectively [30] and molecular dynamics simulations of selected mutations have been carried out [31][32].

**Table 1:** Residues of Significance.

Residue Category (color)	Residues
Identified by Alanine Scanning Mutagenesis (grey) [39]	MDM2: **54** 58 **61** 62 *67* **93**; P53: 19 23 26
Energetically Mutable (purple)	MDM2: 17 29 *65 68 69* 105; P53: 29
Energetically Constrained (red)	MDM2: 19 22 28 37 38 41 43 53 **54** 57 **61** *66* 75 82 85 **93** 97 103 107

**Fig 2:**
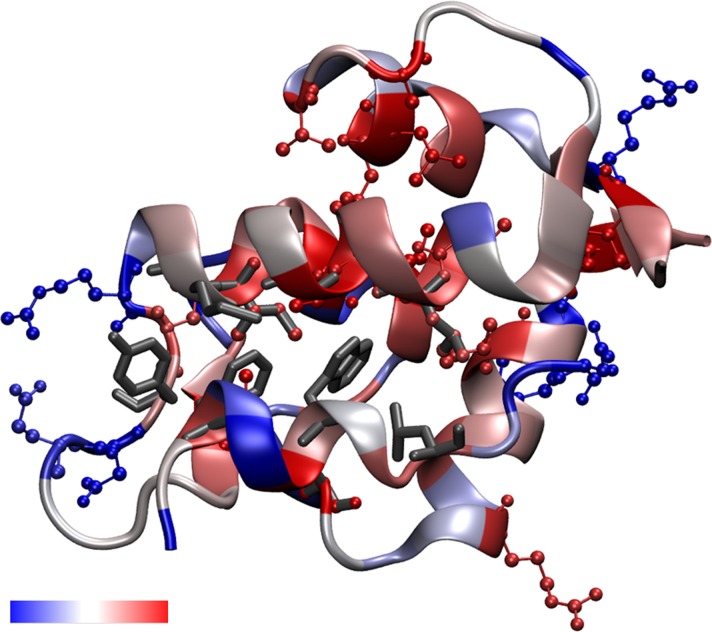
MUMBO energy score for total mutagenesis mapped to the 1YCR crystal structure. The p53 peptide is bound in the foreground. In red and blue ball-and-sticks, significantly energetically constrained and energetically mutable residues are shown, respectively, and are listed in Table 1. Residues shown in sticks are previously identified hot-spots determined through alanine-scanning mutagenesis (listed in Table 1) including the three directly interacting p53 residues. The backbone itself is colored by each position’s z-score, showing the relative constraint of each position along the backbone. The color scale is shown in the accompanying color scale bar, which ranges from energetically mutable (blue) to energetically constrained (red).

The residues of significance identified by experimental alanine scanning and by our exhaustive computational mutagenesis correspond to the residues displayed in Fig 2. Bolded residues are those which were identified both by alanine mutagenesis and MUMBO analysis. Italicized are residues in the area of interest for drug design discussed in the text.

These structural studies led to the development of a series of peptidomimetic inhibitors. In particular, Zhong and Carlson applied the results of their alanine scanning and molecular dynamics to develop a p53 mimetic binding with a calculated free energy of -8.8 kcal/mol as opposed to -7.4kcal/mol for the wild type interaction [39]. The Schephartz group reported the development of beta peptides which, binding with 233 nM affinity, successfully outcompeted a p53 fragment in a competitive fluorescence assay [40]. Th**e** discovery that peptidomimetic structures necessary to inhibit MDM2 can be as short as six residues [39] suggested that small molecules may well be effective also, and the field has witnessed considerable activity in the development of such inhibitors.

A number of structural based studies involving the use of crystallographic structures has yielded considerable insight into the design and improvement of potential therapeutics aimed at disrupting the interface between MDM2 and p53. Recently Popowicz et. al. have critically reviewed these structures [41].

The nutlins [36][42], cis-imadazoline analogs, were the first of the small molecules to be discovered as the result of a screening assay and have been prominent and widespread in anti-cancer studies [42][43][44][8][45]. They exemplify the strategy for structure based drug design for competitive inhibitors targeting the p53 binding site on the MDM2 protein surface. As a point of departure, the initial nutlin structure has been optimized to yield a family of derived inhibitors engineered with a set of two halogens to bind deeply inside of the hydrophobic p53 binding pocket in MDM2, competing with the key native interactions of the p53 peptide Phe^19^, Trp^23^, and Leu^26^ [36]. Nutlin-3 emerged as the most successful with a binding affinity on the order of 30 nM [36] [41]. Subsequent development of other small molecules entailed mimicking this strategy of competitive inhibition targeting the three pockets inhabited by the three native residues, and the location of binding is similar in all structures for which structures are available **(Fig 1B.)**

Similar to the nutlins are the benzodiazepinediones [46], with its most potent family member being TDP222669 at 80 nM, and its crystal structure 1T4E [33] revealing binding in an analogous fashion. Wang et al. describe a set of inhibitors termed spirooxindoles informed by structure based design based on the expansion of the oxindole group to mimic the native Trp23 of the triad, of which the compound MI-888 has an excellent K_i_ of 0.44 nM and showed promise for tumor treatment [47] [48]. Additionally, this proposed inhibitor has been crystalized [34] and has been shown to form a hydrogen bond to Leu54 in MDM2 and to exhibit some induced fit. The imidazoyl-indole family of drugs was developed as part of an attempt to improve upon both the spirooxinodole and nutlin scaffolds [49]. While these compounds were potent with respect to MDMX, they were not as successful as the most optimized spirooxinodole compound [41]. The study demonstrates the use of a drug side chain that binds to residues proximal to the binding site [34] and could be employed as a general strategy to further optimize inhibitors. The chomentotriazolopyrimidines [50] exemplify another class of drugs developed by high throughput screening and again bind in the three pockets with a K_i_ in the submicromolar range [35]. Engineering off this scaffold features a flap which folds over val93 and his96 to increase the number of interactions.

The above five drugs representative examples of each inhibitor class for which a crystal structure has informed design [41]. In addition to these, there are many more which have been developed targeting the same surface, and recent advances have been reviewed [8] [41] [44] [51]. Clearly the challenge of designing potent inhibitors of MDM2 has been met with a plethora of small molecules with a variety of chemistries mainly taking advantage of the three binding pockets utilized by the native interaction [29].

Challenges unveiled at the level of clinical trials or in animal models imply that even the development of tightly binding highly specific inhibitors insufficiently meets the demand for therapies warranting regulatory approval. A recent review article has identified efficacy, drug resistance, and side effects as the three major considerations to take into account during clinical trials [44]. A major concern is the hyperactivation of p53 delocalized from tumors has been observed to enable toxicity in non-cancerous cells [52] [53][54][55]. A further problem arises in that many animal model studies are carried out in mice, yet seemingly subtle sequence differences between human and mouse MDM2 could be sufficient to alter the performance of the drugs and thus may be insufficient to predict the outcome in humans [44]. Poor water solubility of compounds requiring large hydrophobic groups for achieving desired functionality remains problematic for drug discovery in general and is being addressed by research into drug delivery systems [56] continuing to be under development. For example, solubility issues plagued the development of RO-2443 [57], a promising drug operating on the principle of causing sequestration by tetramerization of MDM2 and MDMX.

Given that genomic scale sequencing has provided the insight that cancers arise by the sequential accumulation of mutations conferring a selective growth advantage over time [58], emergence of escape mutations rendering treatments ineffective suggests pre-empting mechanisms of resistance require attention. Rapid acquisition of drug resistant mutations has been shown in to occur with treatment by MDM2 inhibitors and could pose a limit on clinical efficacy [59]. Nutlin-3 has been particularly well studied, and treatment of cell lines with this drug selected for mutations in the DNA binding domain of p53, thus interfering with its ability to specifically recognize target sites and inactivated function as a transcription factor [60] [61] [62]. In addition, mutations on the MDM2 side of interaction emerged from an *in vitro* selection assay involving nutlin-3 [63], suggesting another means of escape mutation acquisition by cancer cells.

Despite the existence of multiple excellent MDM2 inhibitors, these challenges to bringing such small molecules into the clinic will require further development and optimization for properties not necessarily directly related to the binding interface. A major challenge to the field entails diversifying existing small molecules to encompass workarounds for impediments. Ultimately, however, the competitiveness of the inhibitor will need to remain, and the burden of retroactive design will likely take place at the level of molecular design.

In this study, we report on the energy landscape of all possible mutations, indicating which residues are most mutable and which are energetically constrained. Total mutagenesis enabled by computational models of exhaustive point-by-point mutation of the 1YCR crystal structure[15] enables easily quantifiable observations of the energetics intrinsic in the binding interface. Following these determinations, these calculations are used to identify residues on the protein surface that could serve as potential small molecule interaction sites for rational drug design. While many high affinity competitive inhibitors of MDM2 have been developed, diversification of current drugs will likely benefit the development of inhibitors rendering them suitable for clinical use. We examine the MDM2 energy landscape for potential locations of residues unlikely to mutate due to energetic constraints as possible locations for interactions with modified therapeutics.

## Methods

### Crystal Structure Selection

For all modeling and analysis, the crystal structure 1YCR was used [15]. This structure is both representative of the homo sapiens p53-MDM2 binding activity, and is relatively small in structure. To contextualize the location of our identified site for potential drug binding, we selected a sampling of structures, listed in the caption of **Fig 1**.

### Exhaustive “Residue by Residue” Mutagenesis

Performing exhaustive, point mutagenesis of the 1YCR[15] crystal structure required use of the crystallographic refinement program MUMBO[64], which utilizes a template input structure as a scaffold from which a new model can be constructed utilizing the mutated input structure. MUMBO has previously been utilized in order to model and explore T-cell epitope diversity in *Plasmodium falciparum*[65], and protein-protein interface redesign[66]. Performing a mutation can be done algorithmically by the introduction of an additional input file that is designated as the input crystal structure to be refined. Subsequently, a standard rotamerization library is used to determine the optimal rotamerized conformation of each residue including the one added through mutation, and the optimal conformation for each residue is taken to be the most energetically favorable position. MUMBO utilizes the CHARMM force field [67][68] in order to calculate the optimal energetic conformation of each residue. The potential energy is given by:

$$U=\sum_{bonds}\frac{k_{i}}{2}{\left(l_{i}-l_{i,0}\right)}^{2}+\sum_{angles}\frac{k_{i}}{2}{\left(\theta_{i}-\theta_{i,0}\right)}^{2}+\sum_{torsions}\frac{V_{n}}{2}\left(1+\text{cos}\left(n\omega -\gamma \right)\right)+\sum_{i=1}^{N}\sum_{j=i+1}^{N}\left(4\varepsilon_{ij}\left[{\left(\frac{\sigma_{ij}}{r_{ij}}\right)}^{12}-{\left(\frac{\sigma_{ij}}{r_{ij}}\right)}^{6}\right]+\frac{q_{i}q_{j}}{4\pi \varepsilon_{0}r_{ij}}\right)$$ (1)

The total Gibbs free energy for each mutation entails the repacking of all residues of the entire protein using side chain rotamerization on a rigid backbone model. MUMBO sums the optimal conformation energy for each residue in order to assign a total energy (ΔG) for each mutation relative to a side-chain stripped backbone. These values are then standardized by subtracting the WT ΔG determined from a MUMBO simulation of the WT structure, thus yielding ΔΔG of mutation.

$$\Delta \Delta G_{total}=\sum_{residue_{i=17}}^{residue_{i=109}}\Delta G_{residue_{i,mutant}}-\sum_{residue_{i,17}}^{residue_{i,109}}\Delta G_{residue_{i,wt}}$$ (2)

### Structural Visualization and Measurement

Structural models generated through this mutagenic study were visualized in PyMOL [69] or VMD [70]. To view the electrostatic surface potential, the Adapted Poisson-Boltzman Solver was used to generate a model [71]. Charge surface was calculated using the CHARMM force field [67] [68], and was displayed using the red-white-blue charge scheme. All results produced through the use of the Adapted Poisson-Boltzmann Solver were rendered in PyMOL [69].

### Statistical Analysis and Selection of Sites of Interest

The statistical analysis of mutation calculations was conducted by first determining the average the Gibbs free energy of mutation as a function of the twenty possible wild type positions AA over all positions (running from 25 to 109 in MDM2, plus an additional 13 from the p53 fragment) for positions p having the same wild type position in the protein (here handled by Kronecker’s delta to remove non-matching residues from the sum), divided by the total number of amino acids in the protein having the same wild type residue, n_AA._

$$\bar{\Delta \Delta G}\left(AA\right)=\frac{\sum_{p=25}^{p=122}\frac{\sum_{AA=1}^{AA=20}\Delta \Delta G{\left(AA\right)}_{p}}{20}}{n_{AA_{wt}}}\delta_{i,j}\;where\;\left\{\begin{array}{c} \delta =1\;for\;AA_{wt,p_{i}}=AA_{wt,p_{j}}\\ \delta =1\;for\;AA_{wt,p_{i}}\neq AA_{wt,p_{j}} \end{array}\right\}$$ (3)

This yields a 1 x 20 dimensional array holding the average free energies for the twenty wild type amino acid possibilities. At each position, each position specific average ΔΔG was computed as

$$\bar{\Delta \Delta G}\left(p\right)=\frac{\sum_{AA=1}^{AA=20}\Delta \Delta G{\left(AA\right)}_{p}}{20}.$$ (4)

The comparable standard deviations were also computed.

In order to discern if a particular position had a significantly different profile, the position specific ΔΔG(p) was compared to the average plus or minus the standard deviation for its respective wild type amino acid type. If it lied above the average free energy plus standard deviation, then the mutation had a more positive value and was especially constrained, whereas if it lied below, it was more negative and especially mutable. This type of analysis, then, has the effect of assessing if a particular residue, say a certain glycine, happens to have a free energy of mutation which is more unfavorable (or more favorable) than mutations of other glycines in the protein. Additionally, known sites of interest previously identified in the literature by Molecular Dynamics or Alanine-Scanning Mutagenesis [39] were also given detailed consideration, and in fact, some residues met both criteria.

## Results and Discussion

**Fig 3:**
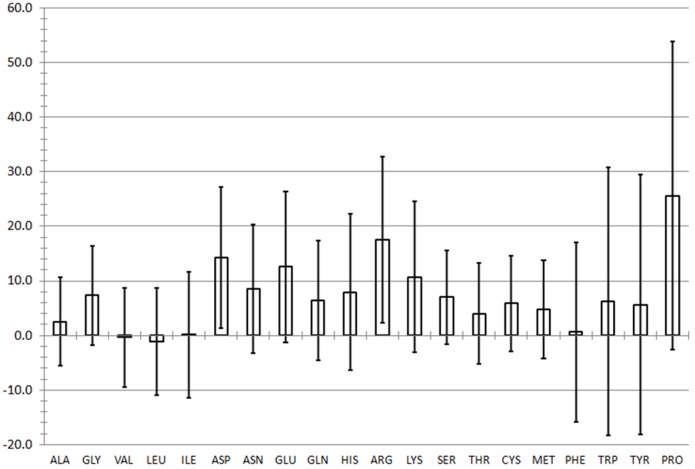
Average MUMBO energy score by wild-type residue type resulting from mutagenesis calculations on the 1YCR crystal structure. Error bars represent 1 standard deviation in average energy.

Total mutagenesis of the 1YCR structure [15] provided a comprehensive energy landscape of the MDM2 protein bound to its cognate p53 fragment. This indicated which positions are highly mutable and which are energetically constrained. **Fig 3** reports the average ΔΔG score obtained for each of the twenty wild type amino acid types being mutated to the other 19 residues. The positive values indicate that mutating the protein is unfavorable in general, as intuitively expected. Furthermore, large aromatic side chains and proline are the most difficult to replace by another residue. Error bars indicate one standard deviation from the average, and any residues at particular positions lying outside the error bars were considered to be significantly mutable (below error bar) or constrained (above error bar). The scores by position have been converted to a color scale, which has been mapped to the crystal structure by residue **(Fig 2)**; the backbone reflects the score of the residue per the scale. According to the criterion of a residue to lie above or below one standard deviation of its respective wild type residue type, six residues which are energetically mutable and nineteen which are energetically constrained were identified **(Table 1)** and are indicated in **Fig 2** with the ball and stick representation, and residues which had been identified by alanine scanning mutagenesis [39] are shown in grey in the licorice representation.

Total mutagenesis analysis has revealed a number of residues which are energetically constrained or mutable. Three residues, 54, 61, and 93 in MDM2, were both identified previously [39] and captured by MUMBO screening; Thus the energetic analysis is in accord with previously reported results. Additional residues which are energetically constrained were identified (**Table 1**), most of which appear within the interior of the protein (**Fig 2**). Conversely, the highly mutable residues (**Table 1**) appear on the surface of the protein. This is consistent with the idea that a densely packed core of a protein would tend to resist packing a new residue to accommodate a mutation, whereas a residue on the surface would be less spatially constrained. In alanine scanning, all mutations except glycine will reduce the side chain size, which is easier to pack than going from a small residue to a larger one due to steric clashes and cavitation energy requirements. In this way, the total mutagenesis differs from alanine scanning, and that more unfavorable positions have been identified concurs with a generalized understanding of protein packing.

The energy scores mapped to the crystal structure can be interpreted to represent the mutability of MDM2 on the basis of energetics. Residues which are energetically constrained are unlikely to form mutants which will persist in a population of tumorigenic cells because the protein will be energetically unstable, disrupting the regulatory feedback between p53 and MDM2. With p53 constitutively in the “on” position as occurred in the case of high doses of MDM2 inhibitors would likely lead to apoptosis, thus eliminating that genotype from the gene pool of developing sarcomas. Thus, our results suggest that a useful strategy for small molecule development to evade drug resistance entails designing inhibitors to interact with residues which are energetically constrained in addition to inhibiting the three key native residues.

**Fig 4:**
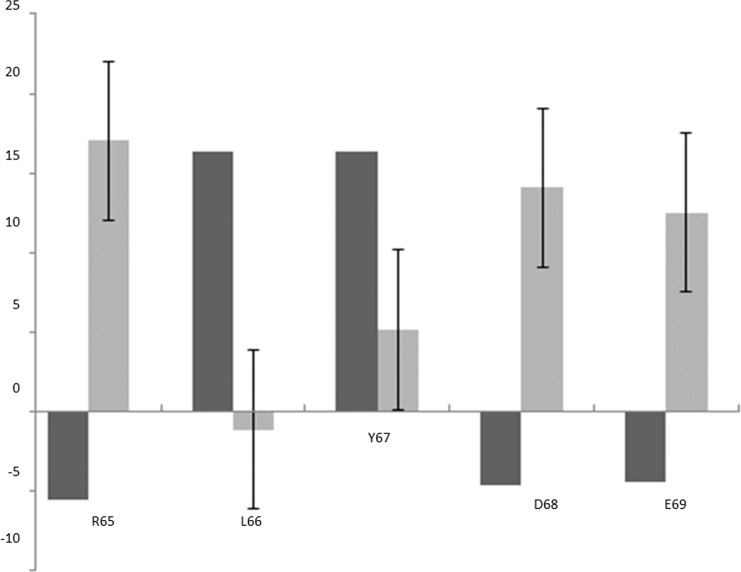
R65 to E69 Tract Average ΔΔG Compared to Average of Respective Wild Type ΔΔG. Average by position, dark grey bars. Average for respective wild type residue as reported in Fig 3 with associated error bars, light grey.

**Fig 5:**
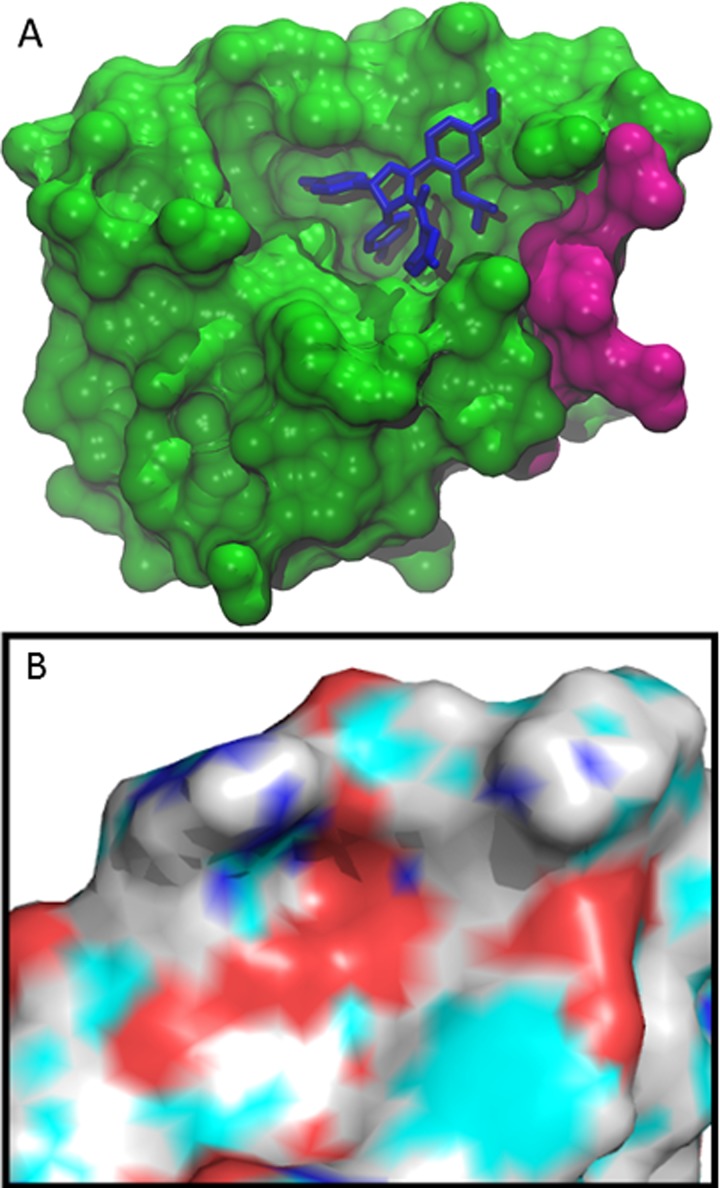
R65 to E69 Tract mapped to MDM2 Crystal Structure. (A) Location of the tract of residues of interest is located near the binding site. Shown here is inhibitor nutlin-3a bound to MDM2 [36]. The energetically significant tract of residues identified, 65–69, is highlighted in magenta. (B) Electrostatic Surface Potential of Identified Site of Interest. This cut out, in the same orientation as (A) and with nutlin removed for clarity, shows the surface charge potential of the range of amino acids that we have identified along with the additional residues that are spatially nearby.

Analysis of the MUMBO-identified sites of interest showed that the R65-E69 tract of residues proximal to the native peptide binding site displayed statistically significant profiles in **Fig 4**, in which the MUMBO score is shown alongside the expected value for its wild type residue type. The location of the tract of residues is shown in **Fig 5A.** L66 and Y67 were identified as highly energetically constrained, suggesting that they would tend not to mutate, as they would destabilize the protein and thus be removed from the population via overactive p53 induced apoptosis. In addition, R65, D68, and E69 emerged as likely to mutate. In order to further characterize this area’s suitability for a druggable site, the electrostatic potential was calculated for the surface of the protein **(Fig 5B.)** The residues of interest lie on the surface of the protein and the identified conserved residues form a hydrophobic patch.

Leading credence to our hypothesis of the importance of these residues, research directed at rationally identifying sites of interest on MDMX, an MDM2 homologue has been carried out and ascertained that V93 and H96 are able to interact with the MI-63 analog [34]. Similar to the tract which we have located, they are proximal to the binding site and demonstrate that designing an inhibitor to interact with surrounding residues is possible to achieve. Interestingly, V93 appears in our results as energetically constrained, suggesting this contact would be less likely to select for escape mutants. The findings of our tract suggest that by targeting L66 and Y67, identified regions of significant energetic constraint, inhibitors could be diversified to interact with these residues with minimal risk of the emergence of escape mutants as a result. However, the high mutability of the electrostatically charged residues suggests that, although developing side chains with higher hydrophilicity to interact with these residues may be attractive to deal with solubility issues, this strategy may select for escape mutants.

## Conclusions

In summary, this study presents the energy landscape of the total mutagenesis of MDM2, which has identified highly mutable and highly constrained sites within the protein. These results may inform how small molecule inhibitors could be diversified in terms of which positions are likely to be resistant to developing escape mutants due to energetic constraints. We have identified a particular tract of residues spanning residues 65 to 69 that display statistically significant energetic properties. Given that a major challenge to curing cancer lies in diversifying existing inhibitors to overcome issues such as escape mutants in order to become clinically useful, suggestions as to how this information may inform such rational design have been presented.
